# Investigating the Role of Solvent in the Formation of Vacancies on Ibuprofen Crystal Facets

**DOI:** 10.1021/acs.cgd.1c01479

**Published:** 2022-04-22

**Authors:** Veselina Marinova, Geoffrey P. F. Wood, Ivan Marziano, Matteo Salvalaglio

**Affiliations:** † Thomas Young Centre and Department of Chemical Engineering, University College London, London WC1E 7JE, United Kingdom; ‡ Department of Materials Science and Engineering, The University of Sheffield, Sheffield S1 3JD, United Kingdom; ¶ Pfizer Worldwide Research and Development, Groton Laboratories, Groton, Connecticut 06340, United States; § Pfizer Worldwide Research and Development, Sandwich, Kent CT13 9NJ, United Kingdom

## Abstract

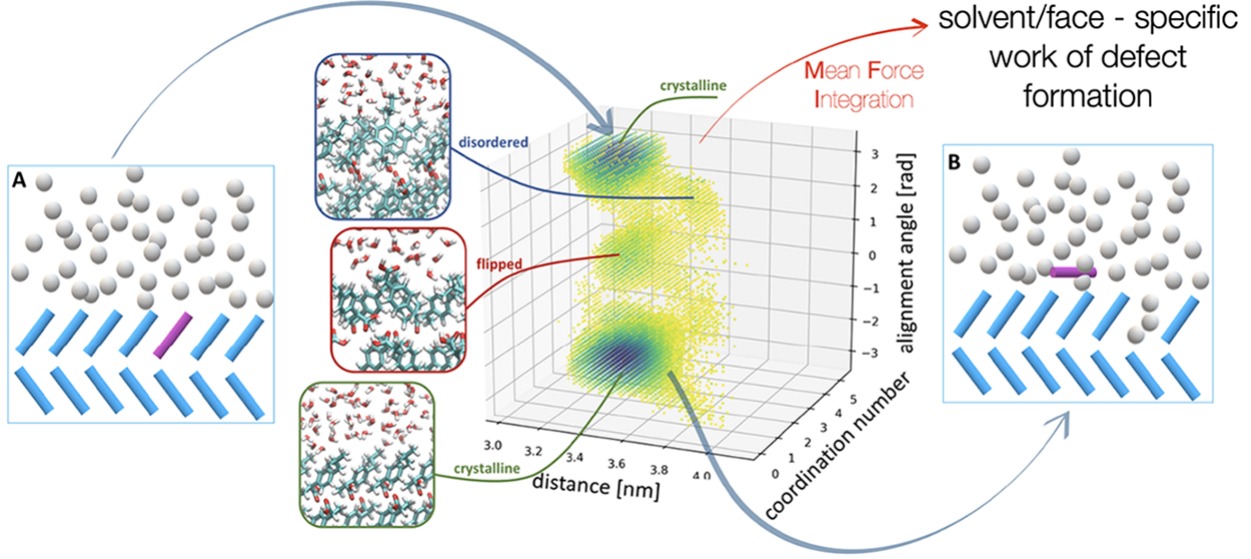

Surface defects play a crucial role in the process of crystal growth, as incorporation of growth units generally takes place on undercoordinated sites on the growing crystal facet. In this work, we use molecular simulations to obtain information on the role of the solvent in the roughening of three morphologically relevant crystal faces of form I of racemic ibuprofen. To this aim, we devise a computational strategy to evaluate the energetic cost associated with the formation of a surface vacancy for a set of ten solvents, covering a range of polarities and hydrogen bonding propensities. We find that the mechanism as well as the work of defect formation are markedly solvent and facet dependent. Based on Mean Force Integration and Well Tempered Metadynamics, the methodology developed in this work has been designed with the aim of capturing solvent effects at the atomistic scale while maintaining the computational efficiency necessary for implementation in high-throughput in-silico screenings of crystallization solvents.

## Abstract 2

Based on Mean Force Integration and Well Tempered Metadynamics, a computational approach to obtain the work associated with defect formation on solvated crystal faces, has been developed. The method is applied here to investigate three faces of the most common ibuprofen polymorph in contact with ten different solvents.

## Introduction

The growth of crystals from solution is inherently affected by interactions of solute molecules with the solvent. These interactions modulate the face-specific growth rates leading to the emergence of solvent-specific crystal morphologies.^1−6^ Understanding how surface–solvent interactions change the morphology at a molecular level is key to the development of rational approaches for the design of crystallization processes.

Examples of solution-controlled crystal morphology alterations have long been of research interest, resulting in an abundance of publications reporting such observations. A landmark paper on the subject by Davey et al.^7^ came out almost four decades ago, looking into the growth morphology of succinic acid upon changing the solvent from water to isopropanol. In their study, the authors identify key solute–solvent interactions, laying the groundwork for understanding solvent-induced crystal morphology. Over the years, a selection of mechanisms have been identified to rationalize the role of the solvent on crystal growth shapes. It is thought that, on certain facets, solvent molecules can act as an impurity, therefore hindering surface diffusion and blocking access to kink sites.^7^ Solvent polarity, steric interactions, and hydrogen bonding capabilities have also been found to affect the growth rate of specific crystal facets,^8,9^ while in some cases Coulomb and van der Waals forces can have a significant impact.^10^

In our recent work,^11^ the dynamics and thermodynamics of solvent molecules at the crystal–solution interface reveal how the type, strength, and lifetime of surface–solvent interactions can have a dramatic impact on the solvent behavior at the crystal surface. Quantifying this information has provided a straightforward and easily accessible screening procedure of identifying solvents which have the potential to affect crystal shape anisotropy. Additionally, an investigation into the thermodynamics and kinetics of solute conformational transitions at the crystal–solution interface has revealed how conformational rearrangements play an important role in the molecular mechanisms of attachment/detachment, display solvent-dependent characteristics, and can affect the kinetics of crystal growth.^12^

The growth morphology of solution-grown crystals, however, is ultimately a direct result of the relative growth kinetics of morphologically dominant facets. Growth and dissolution kinetics of crystal surfaces are governed by the ease of attachment/detachment of solute molecules to/from the crystal, which are processes occurring at defects on the crystal surface.

**Figure 1:**
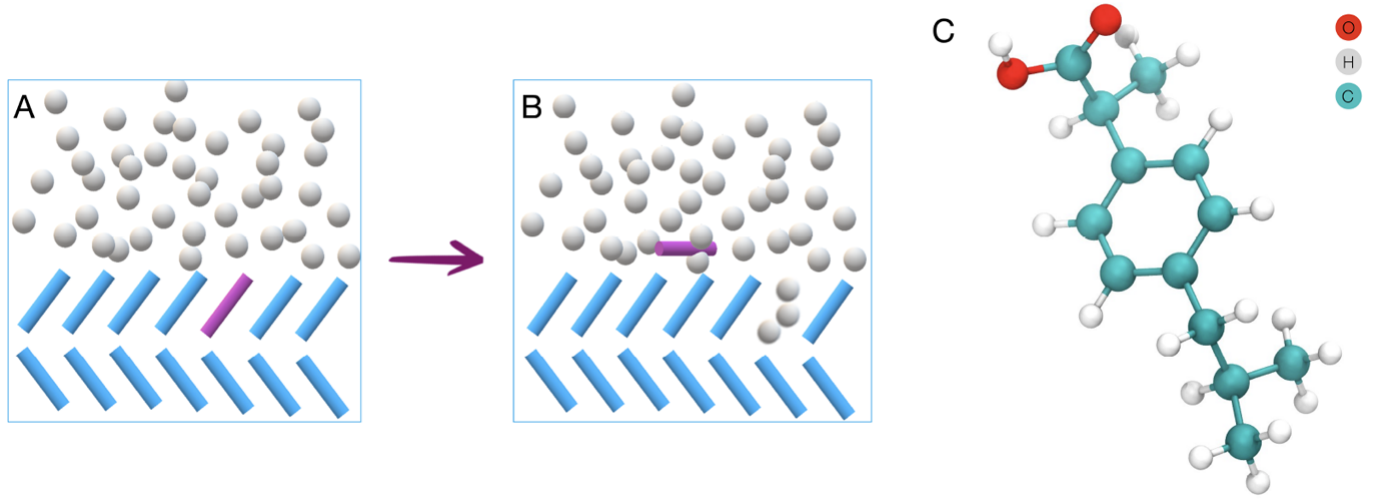
Initial and final state of the process of surface vacancy formation, where state A represents a defect-free crystal surface exposed to the solvent and state B illustrates the final state in which a molecule has detached and can be found adsorbed on the crystal surface. In (C), the molecular structure of ibuprofen is reported.

In this work, we aim to develop and test a computational approach for systematically investigating the formation of point defects at the solid–liquid interface (see Figure 1) and their dependence on the solvent in contact with the crystal phase. The scope of our investigation is to enable large scale screening of different face/solvent combinations in order to gather dynamic information on crystalline–solution interfaces relevant in the design of the solid form of organic crystals. Here we test our approach on ibuprofen crystalline surfaces. In order to bound the parameter space of the computational study, we focus on morphologically dominant crystal facets and model the nucleation of a defect on a flat, defect-free surface. This choice allows to establish a reference process and to avoid developing case studies for all possible types of surface kinks. This in turn enables the definition of a transferable protocol to compare the propensity of defect formation of different surfaces/solvent combinations.

We discuss our results in terms of the interplay between the effect of internal conformational rearrangement and solvent behavior, showing that the detachment rate and the mechanism of the detachment process are heavily face- and solvent-dependent for the majority of the morphologically dominant crystal facets of ibuprofen.

## Methods

In this work, MD simulations, in combination with Well Tempered Metadynamics (WTmetaD), have been used to study the formation of a surface vacancy from a defect-free crystal facet (state A) to an adsorbed on the crystal surface state (state B) as shown in Figure 1. Simulations were performed for each morphologically dominant crystal facet of ibuprofen, namely, the {100}, the {002}, and the {011} faces, for 10 different solvents: water, 1-butanol, toluene, cyclohexanone, cyclohexane, acetonitrile, trichloromethane, methanol, ethyl acetate, and ethanol. The {110} face is occasionally also considered as a morhphologically dominant crystal face, but it was excluded from the analysis in this study due to its generally rough nature and tendency for spontaneous surface roughening and dissolution, indicating a comparably lower barrier to the process of interest and, therefore, lower morphological importance.^11,13^

### System Overview

The system we concentrate on is ibuprofen, or isobutylphenyl propionic acid. Ibuprofen is a commercially available and widely used drug that is member of the nonsteroidal anti-inflammatory pharmaceutical ingredients (APIs).^14^ From a manufacturing point of view, ibuprofen has been identified as an API which crystallizes in different crystal shapes depending on the solvent used during the synthesis process,^15^ which makes it a molecule particularly suited to the objectives of this work. The molecular structure of ibuprofen comprises of a phenyl ring with two *para*-substituents: an isopropyl group and a propionic acid functionality. The molecular structure is characterized by several internal torsional angles, which translate into a moderate degree of flexibility. The aliphatic carbon bonded to the carboxylic acid functionality is a chiral center and so two stereoisomers of ibuprofen exist, known as *S*-ibuprofen and *R*-ibuprofen, where the former is the biologically active form. Until the early 2000s, the only known crystal form of racemic ibuprofen (phase I) was monoclinic ibuprofen with a space group P2_1_/*c*.^16,17^ In 2008, phase II was found through the use of differential scanning calorimetry.^18^ The synthesis of an enantiopure crystal form of *S*-ibuprofen has also been reported in the literature.^19^ Here we consider the most stable form I crystal form of racemic ibuprofen, obtained from the CSD under the deposition code 128796.

### Molecular Dynamics Setup

Molecular dynamics simulations of a slab exposing a dominant crystal facet of ibuprofen, generated with the aid of the functionalities implemented in VESTA^20^ and solvated using the inset-molecules utility as implemented in Gromacs 5.1.4^21^ in each of the 10 different solvents previously mentioned, were performed. The slab thickness was set up so the volume occupied by the crystal is half of the volume occupied by the solvent in order to prevent surface–surface interactions through periodic boundary conditions. The Generalized Amber Force Field (GAFF)^22^ was used to represent system properties. For all systems considered in this work, GAFF is able to reproduce properties consistent with experimental data. In support of this, we report solvent densities in the Supporting Information. Force field parameters for solvent molecules were obtained from the Virtual Chemistry solvent database^23,24^ or parametrized following the standard Amber procedure with antechamber.^25^ A standard cutoff distance of 1.0 nm for the nonbonded interactions was chosen, along with including long-range intermolecular interactions using the Particle Mesh Ewald (PME) approach.^26^ For computational efficiency, a time step of 2 fs was used. Temperature and pressure control have been implemented through the use of the Bussi–Donadio–-Parrinello thermostat,^27^ Berendsen barostat,^28^ and Parrinello–Rahman barostat.^29^

### WTmetaD Setup

Well-tempered metadynamics was used in order to increase the computational efficiency and recover thermodynamic information on the formation of a surface defect for each surface/solvent combination. In this work, the lag time between bias depositions was set up such that the deposition of biasing potential in the transition state ensemble (λ*) is limited, following the protocol discussed by Tiwary and Parrinello.^30^ The biasing protocol was defined via a trial-and-error procedure with the aim of obtaining an optimal balance between computational efficiency and minimal perturbation. The biasing protocol used to reconstruct the free energy profile of the detachment process is reported in Table 1.

**Table 1:** Well-Tempered Metadynamics Parameters Used for the Biasing Protocol for Simulating the Removal of an Ibuprofen Molecule from Morphologically Dominant Crystal Facets

CV	width [rad]	height [*k*_B_*T*]	bias factor [K]	pace [steps]
coordination number	0.15			
distance	0.1	2.5	15	4000
alignment	0.1			

In recovering information for free energy profile calculations, 30 simulations per starting configuration initialized in state A were performed. Each surface/solvent combination was investigated, producing a total of 1320 simulations.

### Collective Variables

**Figure 2:**
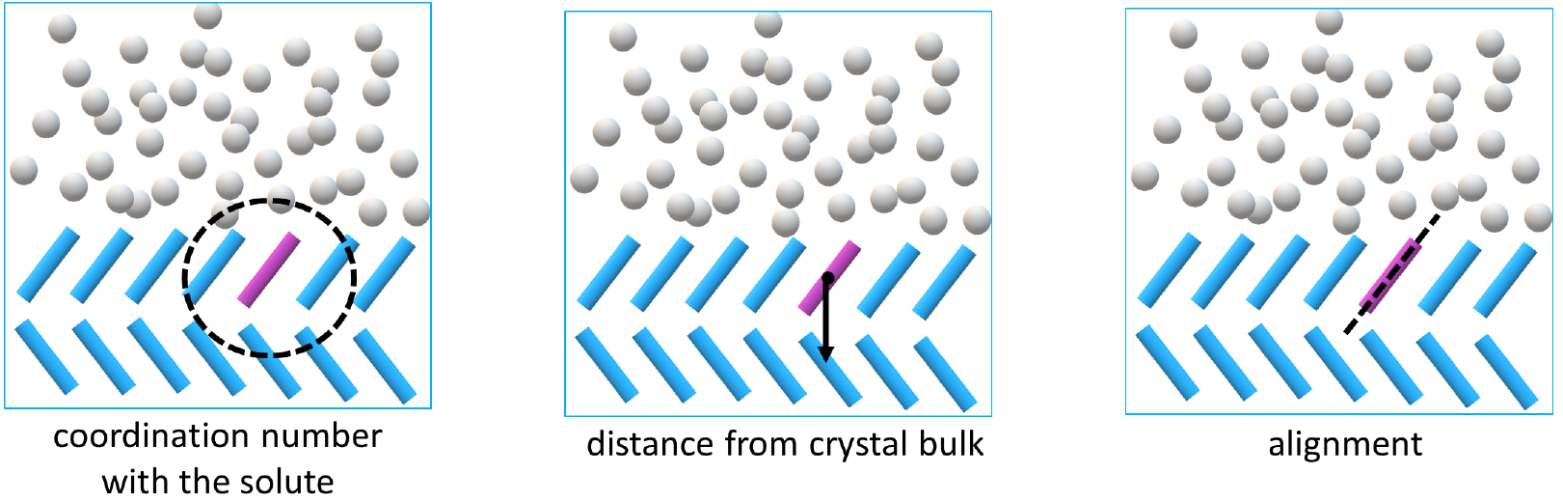
Collective variables used in the process of vacancy formation. From left to right: coordination number of the biased molecule with other solute molecules, distance of the biased molecule from a reference point in the crystal bulk, and degree of alignment of the molecule with respect to its original crystal-like configuration.

WTmetaD was used to enhance the formation of a surface vacancy from each morphologically dominant crystal facet of ibuprofen. To this aim, the external biasing potential was deposited as a function of three collective degrees of freedom so as to be able to distinguish between state A and state B as shown in Figure 1, as well as provide an adequate description of the intermediate states. The collective variables chosen are illustrated in Figure 2.

#### Coordination Number

The coordination number of the biased molecule, within a radius of 0.7 nm, with all other solute molecules was set up in order to monitor the number of solute neighbors within the surface.

#### Distance

The distance between the biased molecule from a reference point within the crystal bulk along the *z*-coordinate, orthogonal to the crystal surface, was set up to monitor the degree of detachment of the molecule.

#### Alignment Angle

An orientation angle which monitors the molecule’s degree of alignment compared to its original crystal-like configuration was also set up. This is an important system descriptor as it allows decoupling of states for which the molecule has lost its crystalline configuration, but is still incorporated into the crystal surface, from the starting configuration. This disorder is found to be important in the molecule removal from the crystal surface and therefore needs to be resolved when applying external biasing potential.

### Mean Force Integration (MFI)

MFI was employed to recover free energy profiles of the formation of surface defects as well as obtain quantitative information on the work performed by the bias potential to generate a surface vacancy. To this aim, a free energy profile was calculated as per eq 1 discussed in the original publication^31^ and shown below:
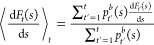
1

In MFI, for each estimate of the mean force, , as well as accounting for the time dependent bias force, , the derivative of the biased probability density *p*_b_(*s*) between every two bias depositions in the time interval [*t*; *t* + τ] is approximated using Gaussian kernel density functions. In the aforementioned time interval, τ refers to the lag time between every two subsequent bias depositions. A bandwidth of *h* = 0.03 was used for the Gaussian kernels and the number of frames used to reconstruct the perturbed mean force term  is *n*_τ_ = 8.

#### Free Energy Calculation

An average time-independent free energy surface ⟨*F*(*s*)⟩ of the process illustrated in Figure 1 was obtained through MFI. For a free energy reconstruction, the simulations set up to recover kinetics from biased sampling were used. These comprise 30 independent replicas per starting configuration, where a starting configuration refers to an ibuprofen molecule embedded in the outermost crystalline layer at the solid/liquid interface for each surface/solvent combination.

#### Work Calculation

The work performed by the biasing protocol in removal of a molecule from the crystal surface was calculated for each surface/solvent combination. This information is obtained by calculating the quantity:
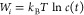
2where *c*(*t*) is defined as
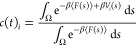
3where *i* is the number of the simulation performed and *V*_*i*_(*s*) is the total bias accumulated for the duration of the simulation. The work reported in subsequent sections refers to the average value of *W*_*i*_ computed over the set of simulations performed for a single system. The estimate of *W̅* is robust with respect to the simulation setup, and it has been used to evaluate the solvent-dependence on the thermodynamics of the defect formation for all crystal facets studied.

## Results

Our aim in this work is to obtain information on the thermodynamics of surface defect formation on each of the morphologically dominant crystal facets of ibuprofen in the presence of different solvents. We analyze the apolar {100}, polar {100}, {011} and {002} faces and analyze the complexity of the detachment mechanism associated with defect formation. Using MFI, we obtain a free energy profile of the process of formation of a surface defect, represented in Figure 1 as a transition between the states labeled as A and B, corresponding to a smooth surface, and a surface presenting a single surface vacancy to the solution environment. In order to investigate how differences in the environment, i.e., the specific surface and the solvent affect the mechanism of the detachment process, we also characterize the complexity of the detachment mechanism and the quality if the collective variables used to describe the process by critically analyzing the distribution of transition times obtained from metadynamics simulations.

### Free Energy Calculations

To gain insight into the thermodynamics associated with the removal of molecule from the crystal surface, a free energy landscape for each surface/solvent pair is calculated from a number of independent WTmetaD simulations using Mean Force Integration. By implementing a general approach rooted in Thermodynamic Integration, MFI allows us to consistently use sampling obtained in independent biased simulations to estimate a joint FES, as discussed in ref (31). The FES describes the free energy associated with a detachment event and the basins recovered in CV space correspond to high probability configuration visited in the pathway toward detachment. The FES are converged up to the barrier associated with detachment from the surface.

In order to assess the internal consistency and reliability of the method in this specific application, a free energy profile is calculated for each of the four biasing strategies outlined in Table 2 for the case of an ibuprofen detachment from the {100} apolar crystal face in the presence of water. Table 2 also reports the average difference between any two free energy surfaces calculated, as well as the standard deviation of that mean. The maximum average difference between any two cases is found to be just under 2*k*_B_*T*, with a standard deviation within 0.5*k*_B_*T*. These results demonstrate a consistent reconstruction of the FES associated with the removal of a molecule from the crystal surface, obtained from different biasing protocols.

**Table 2:** Consistency of the Free Energy Calculation across WTmetaD Protocols for the FES Associated with Defect Formation on the {100} Apolar Crystal Face in Water Reconstructed Using MFI^a^

	1	2	3	4
	γ = 20, τ = 4	γ = 15, τ = 4	γ = 13, τ = 5	γ = 10, τ = 5
1	–	2.7	4.3	4.6
2	*0.5*	–	1.6	1.8
3	*1.4*	*1.0*	–	0.5
4	*1.2*	*1.1*	*0.8*	–

a Both the average difference  and the standard deviation of the difference σ_δ*F*_ between every pair of FES biasing protocols tested (1-4) are reported in the table. The average deviation  is reported in the upper right triangle (normal text), the standard deviation σ_δ*F*_ in the lower left triangle (italicized text). Labels 1-4 indicate different WTmetaD biasing protocols varying bias factor (γ, [−]) and pace of Gaussian deposition (τ, [10^3^ MD steps]).

**Figure 3:**
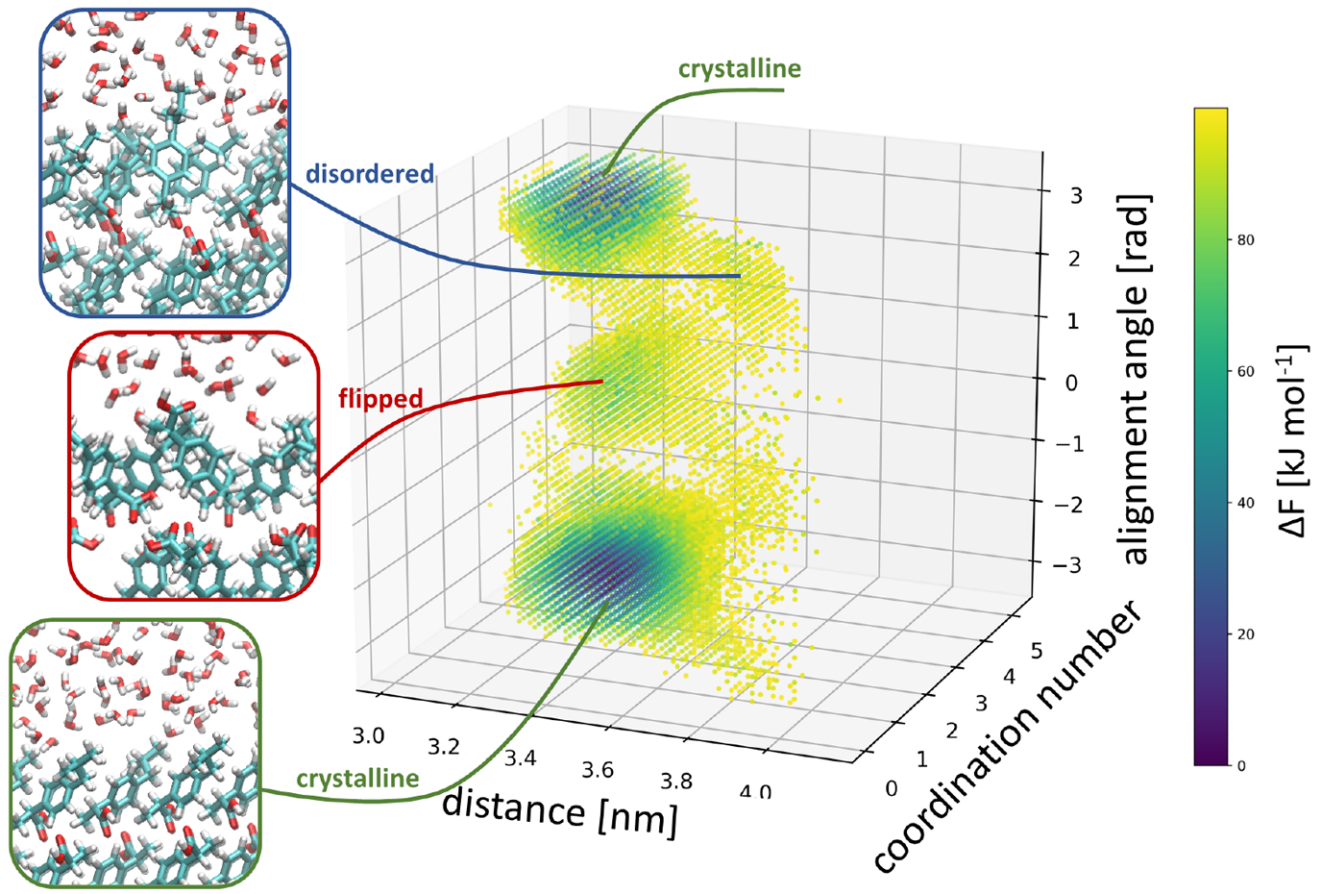
Free energy surface associated with the formation of a surface defect on the {100} apolar crystal facet in water. Starting configuration is shown in green, where the molecule is in a crystalline configuration. Transition states, in some cases, are observed to contain a disordered molecule within the crystal surface (blue) and a flipped configuration where the molecule is aligned as per the crystalline configuration; however, the opposite para substituent is exposed to the solvent (red).

An example free energy surface for the case of a molecule removal from the {100} apolar crystal surface in water for biasing protocol no. 2 is shown in Figure 3. The free energy is dominated by the starting configuration of a perfectly aligned solute molecule incorporated into the crystal surface which can be found at a low distance, high solute coordination number, and alignment angle of ±π, referred to as the crystalline configuration in Figure 3. The value of the angle here is arbitrary with respect to how the reference has been defined. This configuration accounts for 99.5% of the probability distribution of states in the reactant basin. On the given free energy profile, a local minimum at an angle of 0 rad can be identified, which corresponds to a configuration in which the molecule has flipped orientation within the crystal surface and the opposite *para*-substituents of the aromatic ring are exposed to the solution, while the overall alignment of the molecule is crystal-like. This configuration accounts for 0.04% of all microstates within the reactant basin for this particular setup. The remaining 0.01% of configurations within the basin account for intermediate states for which the molecule has lost order within the crystal surface but is not fully detached, labeled as disordered state in Figure 3.

### Detachment Mechanism Investigation

In previous work,^11^ we demonstrated how surface–solvent interactions have the ability to affect the surface roughness as well as the solvent behavior at the crystal surface, which in turn will play an important role in the processes of incorporation or detachment of growth units from the crystal surface. Moreover, internal conformational rearrangements of the solute are typically present and can be dependent on both the crystal face and the solvent. The combination of these effects has an impact on the mechanism of detachment and on the role of conformationally or orientationally disordered configurations along that pathway. To understand more about the role of the noncrystal configurations found through the analysis of the FE profile, illustrated in Figure 3, the detachment trajectories are investigated in more detail.

**Figure 4:**
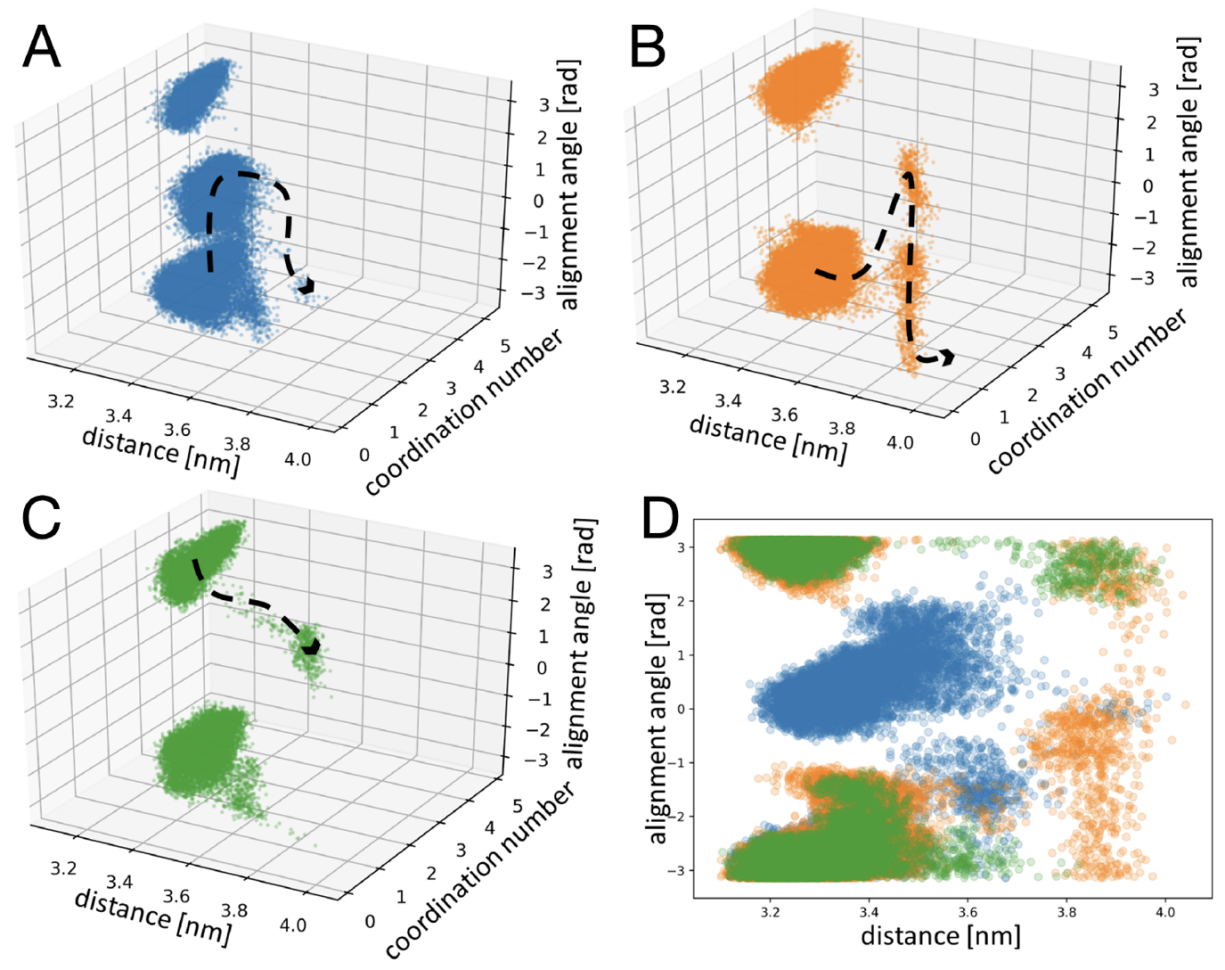
Sampling region for three different trajectories of a molecular detachment from the {100} apolar surface in water in collective variable space. In green, a direct detachment from crystalline state out of the crystal slab along the distance and coordination number coordinate is illustrated. A detachment mechanism involving rearrangement of the molecule within the crystal surface before detachment is illustrated in blue and orange, where the former depicts a particular case of mechanism going via a flipped configuration as shown in Figure 3.

The analysis of the trajectories obtained for all surface/solvent combinations reveals three broad groups of detachment mechanisms occurring, illustrated in Figure 4. In a fraction of trajectories, the process occurs via a linear path of detachment, represented by a simultaneous increase of the distance of the molecule from its lattice site and loss of coordination with other solute molecules as shown in Figure 4 in green. This process can occur *either with or without* a significant change in the relative orientation of the molecule within its crystalline state.

In particular, the second subset of trajectories reveals a process for which a single molecule detaches via a *flipped* configuration as illustrated in Figure 4 in blue. Trajectory analysis reveals that a flipped intermediate configuration is observed on average in 10% of the detachment events; however, the observation of this mechanism is markedly surface and solvent dependent. The highest occurrence of detachment events following this mechanism is observed in water, where more than 80% of the trajectories follow this pathway. For some surface/solvent combinations, for example, {100} apolar in ethanol or {002} in toluene, this mechanism is never observed. This observation shows how the solvent affects the behavior of a growth unit upon removal from the surface, practically altering the defect formation mechanism.

A third, and more frequent mechanism encompasses detachment trajectories for which the molecule does not follow a linear path in CV space, but rather the molecule explores a range of configurations in what was referred to previously as the disordered state (see Figure 3), an example of which is shown in Figure 4 in orange. This detachment mechanism, occurring via a disordered state, can be preceded by either a crystalline or a flipped starting configuration. The probability of observing a detachment mechanism via an intermediate disordered state, as shown in Figure 4 in orange, also varies upon changing the solvent and can be observed in approximately 30% of the trajectories.

**Figure 5:**
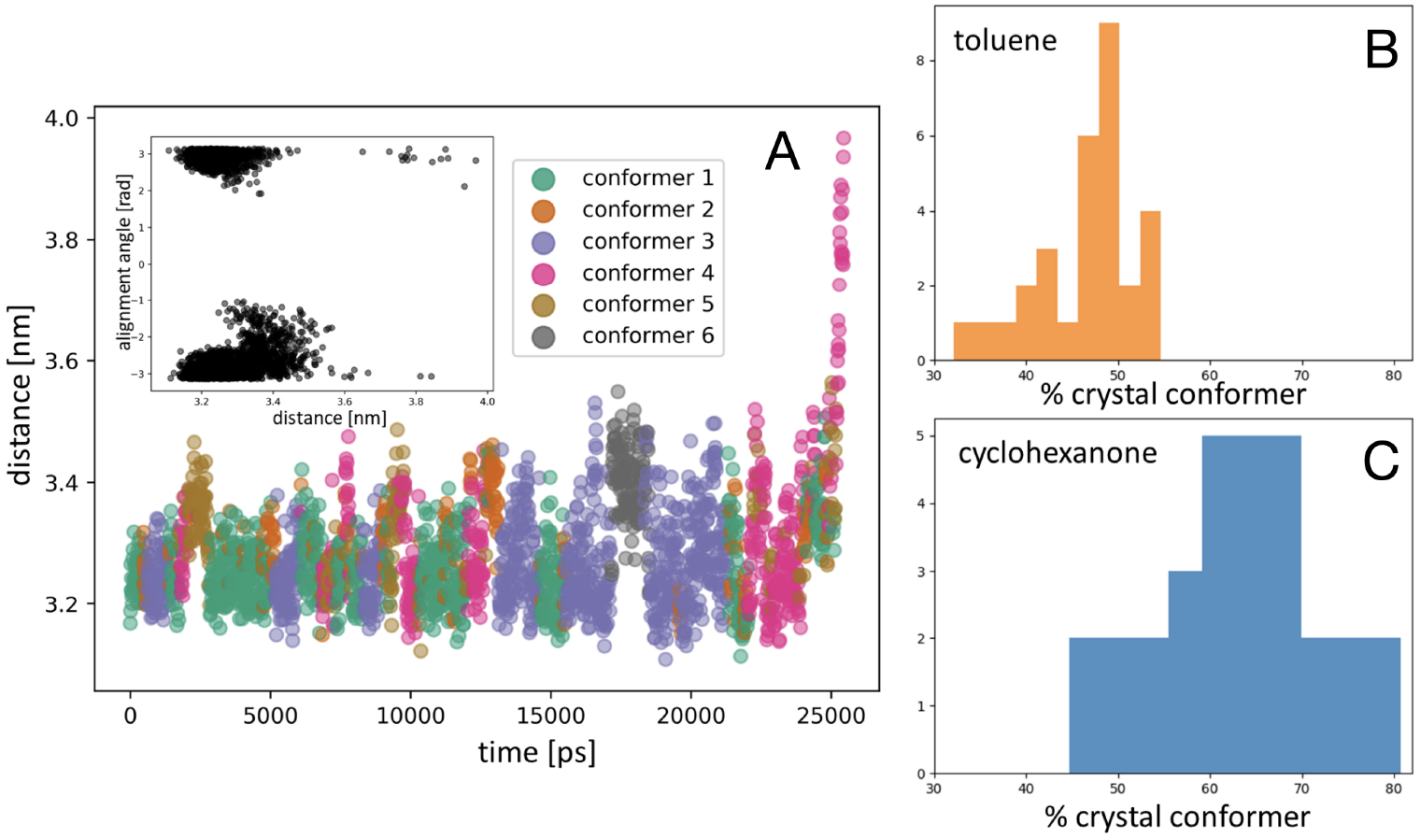
(A) Trajectory displaying a vacancy formation as a function of time and distance for a linear detachment mechanism for the {100} apolar surface in cyclohexanone. (B,C) Histograms of the percentage of crystal-like conformer (conformer 1) observed along a detachment trajectory from the {100} apolar surface in two solvents: toluene (B) and cyclohexanone (C).

Further to the discussion on the possible detachment mechanism, an investigation into the internal conformational rearrangement within the detaching molecule is carried out. This analysis reveals that conformational rearrangements occurring are diverse and inconsistent when comparing between simulations within the same setup group. As an example, a typical trajectory of time vs distance is shown in Figure 5, where color represents the conformation, adopted by the detaching ibuprofen molecule, following the nomenclature proposed by Marinova et al.^12^

The trajectory chosen here is an example of the simplest form of detachment following a linear path in CV space without a change in the alignment angle. Even for this path of defect formation, conformational analysis reveals that a lot of internal conformational transformations are occurring. This observation is not surprising as the conformational population of ibuprofen is state-dependent and so detachment is likely to be coupled with internal conformational rearrangements.

The analysis here supports the hypothesis made in the previous section of the presence of multiple mechanisms, associated with a formation of a surface defect for particular surface/solvent combinations, disrupting the overall distribution of transition times. In reality, it is likely that the presence of multiple mechanisms, coupled with internal configuration, is the reason why the transition state ensemble is in some cases perturbed, leading to poor statistical validation.

### Work Associated with a Surface Molecule Removal

**Figure 6:**
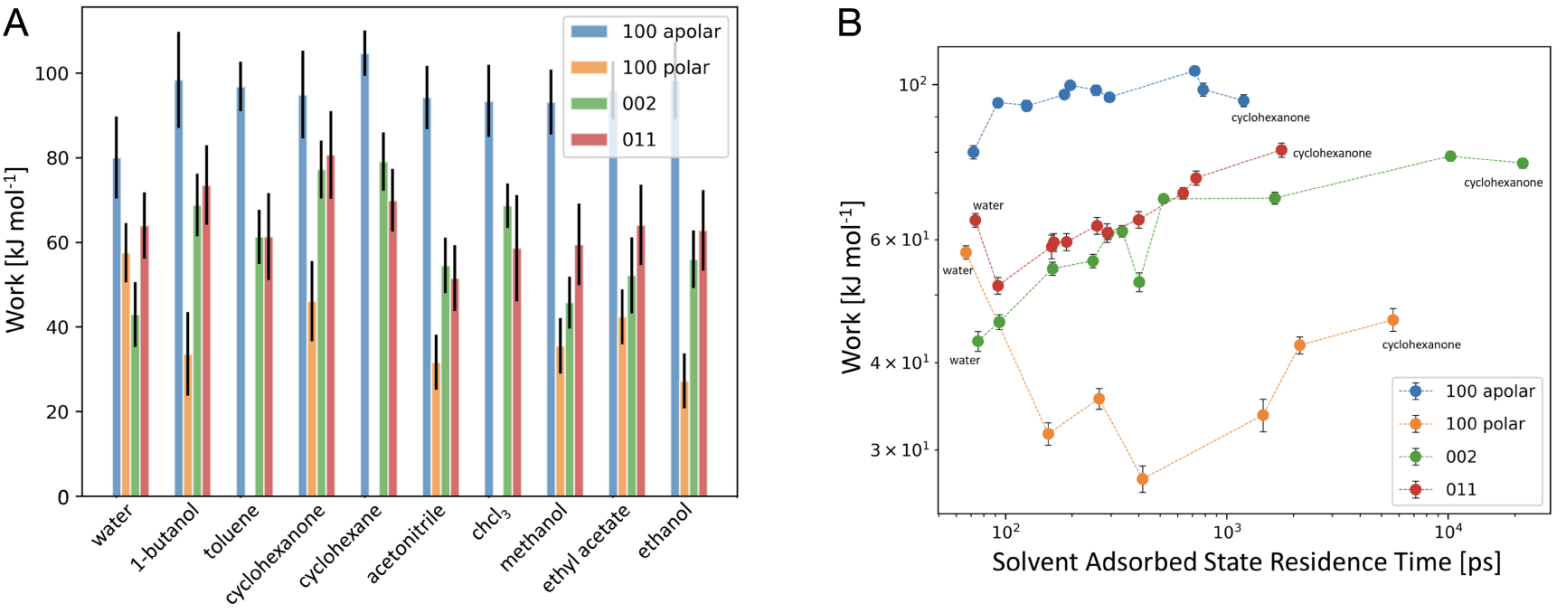
Defect formation of at the crystal/solution interface. (A) Work performed by the biasing algorithm to induce the formation of a surface vacancy for each surface/solvent combination. Error bars represent the standard deviation of the Work computed over 30 repetitions of the detachment process for every solvent/surface combination. (B) Solvent residence time at the crystal surface [ps] vs work [kJ/mol] necessary for the removal of a molecule from a defect-free ibuprofen crystal facet.

The work performed by the biasing potential for the formation of a surface defect was calculated for each surface/solvent combination in order to extract quantitative thermodynamic information for the process. The findings for each case are reported in Figure 6A. Overall, the absolute value of the work associated with a removal of a surface molecule is dominated by the crystal surface morphology. In particular, the formation of a surface vacancy is least probable on the {100} *apolar* crystal facets in all solvent cases, while the probability is found to be highest on its *polar* counter layer. These trends correlate with the observations made in the discussion of the transition times in the previous section, suggesting the presence of intrinsic face-dependent factors controlling the absolute process kinetics and thermodynamics. At the same time, it can be noted that while the values for the work for the case of the {100} *apolar* crystal face vary only marginally, those calculated for the rest of the crystal facets exhibit a much more significant variation when changing the solvent. This observation indicates that the polar {100}, {002} and {011} crystal faces are much more prone to specific-surface solvent interactions, subsequently affecting the ease of molecule association/dissociation at the crystal surface.

In a previous work,^11^ we investigated in detail the residence time of different solvents at morphologically relevant faces of ibuprofen. The work of defect formation obtained here is reported as a function of the solvent residence time at the crystal–solution interface computed in ref (11), with the aim of evaluating the extend of correlation between these two quantities.

Assessing Figure 6B, a prominent correlation between the residence time of a solvent molecule at the crystal surface and the work required to form a surface vacancy can be noted, particularly for the {002} and {011} crystal facets. This relationship indicates that the mechanism of detachment for these surfaces is strongly affected by desolvation. We note that, for these two faces of ibuprofen, the relative dissolution rate was recently measured,^32^ showing the two faces dissolve with rates of the same order of magnitude. This is consistent with our calculations that identify very similar works of defect formation for these two facets across all solvents (see Figure 6A).

The case of the apolar {100} crystal facet differs from the {002} and {011} facets. Here little correlation between work and solvent residence time is present, and it can be concluded that the processes of surface integration and removal of a growth unit would be the rate-determining step in the processes of growth and dissolution, respectively. Furthermore, high work of defect formation, weakly dependent on the solvent residence time, suggests that when present the apolar {100} face would present a low density of surface defects. On the other hand, the {100} polar surface, which exhibits opposite behavior (low work of defect formation, strongly solvent dependent), when present is likely to exhibit high density of surface defects.

Finally, the fact that across all cases examined the of solvent dynamics correlates with the energetic cost of defect formation indicates the importance of explicitly including solvent degrees of freedom in the mechanistic description of molecular processes at the crystal/solution interface.

## Conclusions

In this work, we have investigated the formation of a surface vacancy defect on three morphologically dominant crystal facets of ibuprofen for a set of 10 different solvents. This study enables a general approach of recovering thermodynamic information on the removal of a surface molecule, which provides a measure of the face and solvent specific tendency of roughening during crystallization, yielding information on the ease of crystallization and shape anisotropy. In particular, we reveal how assessing the correlation between the work associated with the formation of a surface vacancy with the solvent residence time at the crystal surface is indicative of the rate-determining step in the process of growth and dissolution at the specific crystal facets. The protocol for calculating the work of defect formation *W̅*, outlined in this work, can be easily implemented within the context of large scale virtual solvent screenings for the rational design of crystallization processes.

## Supporting Information Available

Surface/solvent combinations considered in this work; experimental solvent density at ambient conditions; internal torsional angles of ibuprofen used to describe the conformational rearrangement of the molecule (PDF)
