# Supplementary material for: Investigating the Role of Solvent in the Formation of Vacancies on Ibuprofen Crystal Facets
Source: Cryst Growth Des. 2022 Apr 22;22(5):3034–41. doi: 10.1021/acs.cgd.1c01479 (PMC9073942; doi:10.1021/acs.cgd.1c01479)
Supplement: Supplementary file 1 — cg1c01479_si_001.pdf [file cg1c01479_si_001.pdf]

Investigating the Role of Solvent in the Formation of Vacancies on Ibuprofen Crystal Facets
Supplementary Material

Veselina Marinova,[†] Geoffrey P. F. Wood,[¶] Ivan Marziano,[§] and Matteo Salvalaglio[*,†]

†*Thomas Young Centre and Department of Chemical Engineering, University College London, London WC1E 7JE, UK.*

‡*Department of Materials Science and Engineering, The University of Sheffield, Sheffield S1 3JD, UK*

¶*Pfizer Worldwide Research and Development, Groton Laboratories, Groton, Connecticut 06340, USA*

§*Pfizer Worldwide Research and Development, Sandwich, Kent CT13 9NJ, UK*

E-mail: m.salvalaglio@ucl.ac.uk

Bulk Solvent Analysis

This section reports the density at ambient conditions (298.15 K and 1 bar) for all solvents used in this study obtained from MD simulations of the solvent bulk, along with a comparison to the experimental reported density for each (Table S1). Experimental values of the density were obtained through the open chemistry database PubChem at the National Institutes of Health, part of the US Department of Health&Human Services.

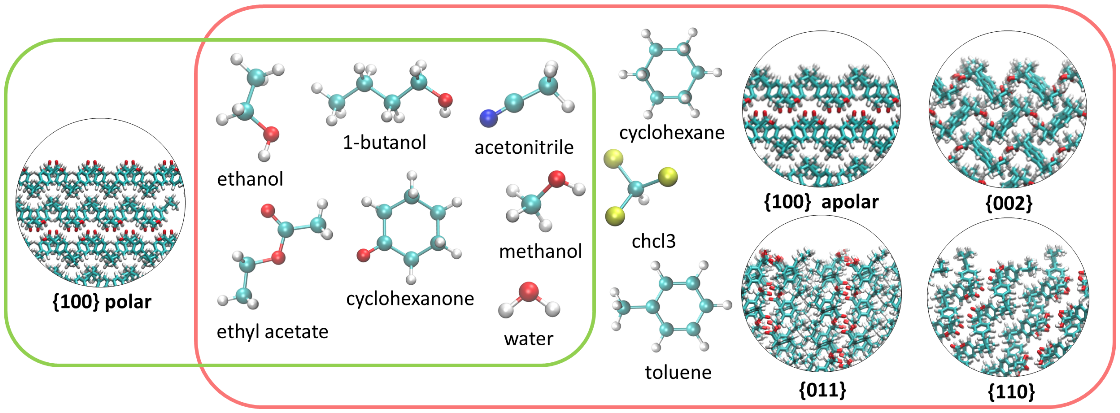

Figure S1: Surface/solvent combinations considered in this work, figure reproduced from Ref.[1] The pink enclosure shows that each of the surfaces apolar {100} , {002}, {011} and {110} is combined with each of the solvents shown, whilst the green enclosure shows the solvents used in combination with the polar {100} surface. All images have been created with VMD[2]

Table S1: Experimental solvent density at ambient conditions alongside values obtained from MD simulations of the bulk solvent. The percentage error of the MD result with respect to the experimental reference is also listed.

**solvent**	**density [kg m^3]**		
	experimental	MD	% error
water	997	985	1.5
1-butanol	810	814	0.5
toluene	867	849	2
cyclohexanone	948	935	1.4
cyclohexane	779	766	1.7
acetonitrile	787	719	8.5
trichloromethane	1483	1406	5.6
ethyl acetate	902	930	3.1
methanol	792	804	1.5
ethanol	790	786	0.4

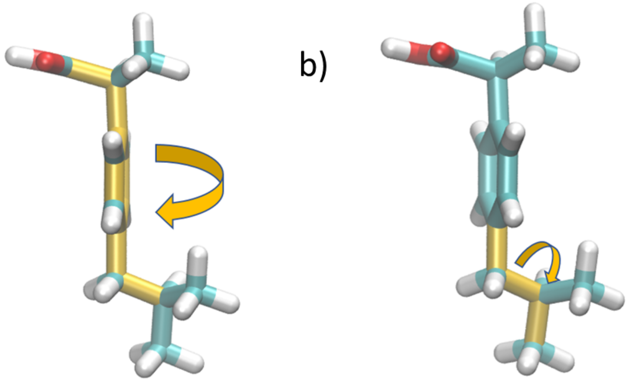

Figure S2: Internal torsional angles of ibuprofen used to describe the conformational rearrangement of the molecule, figure reproduced from Ref.[3] a) Global torsional angle describes the rotation of the *para*-substituent groups around the vertical axis of the molecule and b) Local torsional angle describes the degree of interchange between the methyl groups of the *iso*-butanyl substituent.

References

(1) Marinova, V.; Wood, G. P.; Marziano, I.; Salvalaglio, M. Solvent Dynamics and Thermodynamics at the Crystal-Solution Interface of Ibuprofen. *Crystal Growth and Design* **2019**, *19*, 6534–6541.

(2) Humphrey, W.; Dalke, A.; Schulten, K. VMD: Visual molecular dynamics. *Journal of Molecular Graphics* **1996**, *14*, 33–38.

(3) Marinova, V.; Wood, G. P.; Marziano, I.; Salvalaglio, M. Dynamics and Thermodynamics of Ibuprofen Conformational Isomerism at the Crystal/Solution Interface. *Journal of Chemical Theory and Computation* **2018**, *14*, 6484–6494.
